# Scratching the surface: biomarkers and neurobiomarkers for improved allergic contact dermatitis management

**DOI:** 10.3389/falgy.2025.1564528

**Published:** 2025-03-13

**Authors:** Akimi Sasaki, Manuel Sargen, Anish R. Maskey, Xiu-Min Li

**Affiliations:** ^1^ Department of Pathology, Microbiology, & Immunology, New York Medical College, Valhalla, NY, United States; ^2^ Department of Otolaryngology, New York Medical College, Valhalla, NY, United States; ^3^ Department of Dermatology, New York Medical College, Valhalla, NY, United States

**Keywords:** neurobiomarkers, pruritus, inflammation, IL-31, allergic contact dermatitis

## Abstract

Allergic contact dermatitis (ACD), also known as allergic eczema, is a common inflammatory skin disorder that affects millions of Americans and imposes significant physical, psychological, and economic burdens. Differentiating ACD from other forms of dermatitis remains a challenge, with patch testing as the gold standard. Despite its utility, patch testing can lack diagnostic accuracy, highlighting the importance of molecular biomarkers to refine diagnosis and treatment. Advances in transcriptomics and machine-learning have enabled the identification of biomarkers involved in ACD, such as loricrin (LOR), ADAM8, CD47, BATF, SELE, and IL-37. Moreover, biomarkers such as LOR, NMF, and TEWL, may have prognostic value in evaluating therapeutic response. Emerging neurological biomarkers (neurobiomarkers), including IL-31 and TRPV1, target pathways involved in the pruritic and inflammatory responses, offering novel therapeutic targets as well. This mini review summarizes current ACD treatments, biomarkers for targeted therapies, and emphasizes the role of neurobiomarkers in ACD treatment. Additional research on the validity of the therapeutic potential of these biomarkers is necessary to improve ACD treatment and outcomes.

## 1 Introduction

Allergic contact dermatitis (ACD) or allergic eczema, is a prevalent inflammatory skin disorder characterized by pruritus, erythema, vesicles, and scaling of the skin in response to allergen exposure (1). It is a type IV delayed hypersensitivity reaction that affects millions of Americans and accounts for a substantial proportion of dermatological consultations, with some studies estimating a prevalence of 20% in the general population (1). ACD can result in notable physical disabilities and lost workdays, contributing to significant reductions in quality of life and increased financial burdens (2).

Despite advancements in understanding ACD, differentiating it from irritant contact dermatitis (ICD), a nonallergic skin reaction that does not involve sensitization or immunological memory, and other types of dermatitis, remains a challenge (3). Current diagnosis of ACD relies on patch testing as the gold standard, complemented by an assessment of clinical presentation and exposure history (2, 3). Despite its clinical utility in identifying allergens, patch testing is reliant on subjective interpretations and may be insufficient in differentiating ACD and ICD due to overlapping clinical presentations (2).

Furthermore, while ACD and ICD act through different mechanistic pathways, treatment is approached similarly. Management emphasizes allergen or irritant avoidance with supplemental topical treatments, corticosteroids, phototherapy, and systemic immunosuppressants for severe cases (3). Consequently, there is a need for molecular biomarkers to distinguish ACD from ICD to provide targeted therapies.

This review explores current treatment approaches to ACD, discusses their limitations, and examines emerging biomarkers with therapeutic potential. In addition, given the limited scientific literature on neurological biomarkers (neurobiomarkers) involved in ACD-related pruritus, this review highlights some potential neurobiomarker targets for precision therapy in improving outcomes for patients with ACD.

## 2 Pathophysiology of ACD

**Figure 1:**
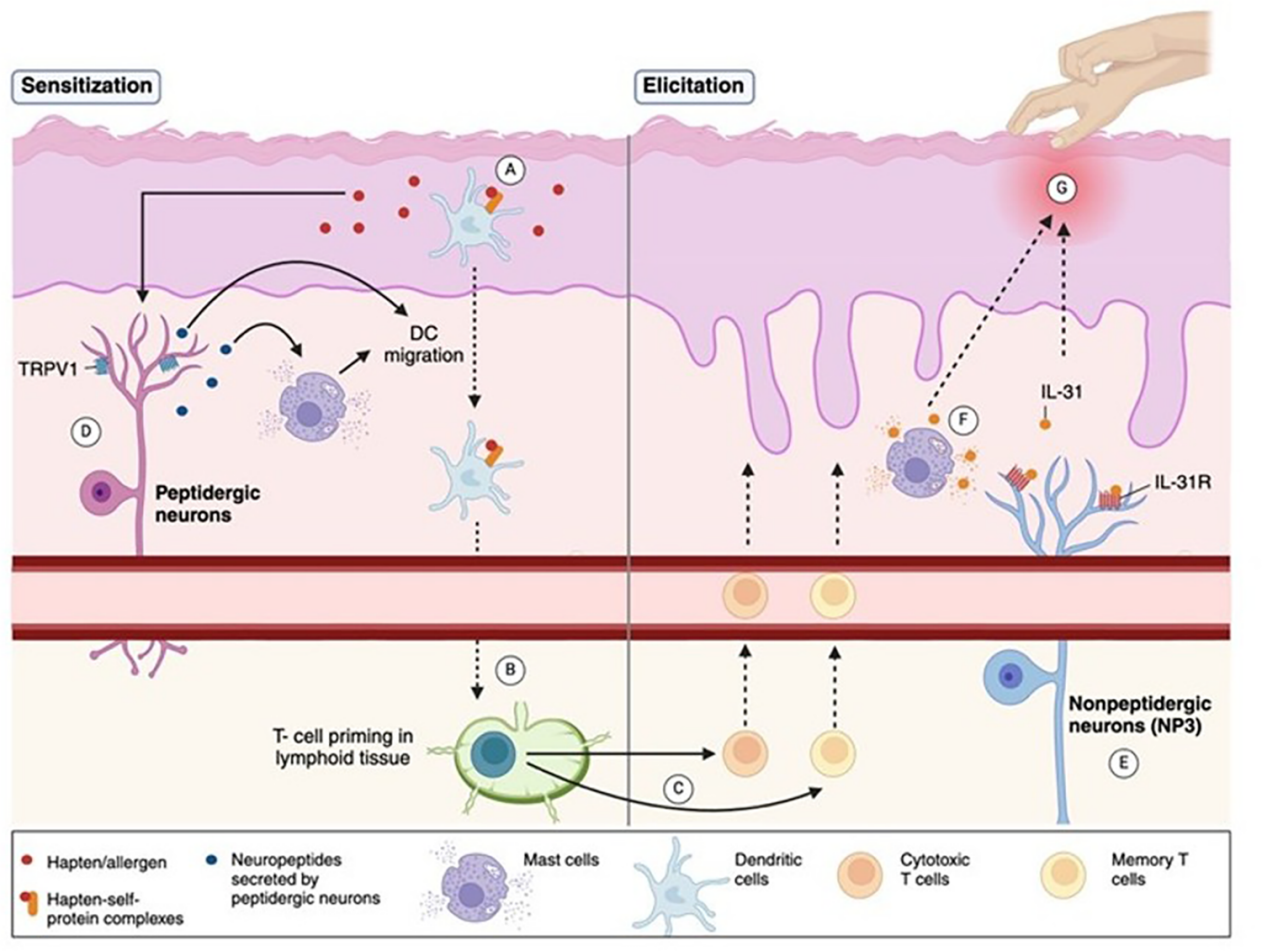
Pathophysiology of ACD and associated neuro biomarkers. The pathophysiology of ACD involves a two-phase immune response. **(A)** In the sensitization phase, allergens/haptens penetrate the skin, form hapten-self-protein complexes, and are processed by dendritic cells (DCs), promoting T-cell priming in lymphoid tissue. **(B,C)** The elicitation phase occurs upon re-exposure to the allergen, activating effector and memory T-cells that migrate to the skin, causing inflammation, erythema, or spongiosis. **(D)** Peptidergic neurons sustain inflammation by releasing neuropeptides that activate mast cells, promote DC migration, and enhance T-cell priming. TRPV1, an ion channel expressed on peptidergic neurons, contributes to inflammatory signaling. **(E)** Nonpeptidergic neurons, especially the NP3 subset, express the IL-31 receptor (IL-31R) complex, and are activated during allergen re-exposure to maintain itch perception. **(F)** IL-31, a pruritogenic cytokine, is secreted by activated T-cells and mast cells, amplifying itch and inflammation. **(G)** The combined immune and neural pathways drive the clinical features of ACD, namely chronic itch. (Created with BioRender).

The pathophysiology of ACD involves a complex interplay between immune mechanisms, epidermal barrier dysfunction, and neuroimmune interactions. ACD is classified as a delayed type IV hypersensitivity reaction following topical exposure to sensitizing agents (2). It is mediated by the activation of allergen-specific T cells (1) and involves of both the innate and acquired immune responses (3). The immune response to ACD involves two phases. The first phase is the sensitization phase, in which the immune system is primed by the allergen. During this phase, allergens penetrate the skin and lead to the formation of hapten-self-protein complexes and are processed by dendritic cells (DC) (Figure 1A), leading to T- cell priming in lymphoid tissue (Figure 1B) (3). The subsequent elicitation phase is triggered by re-exposure to the allergen, in which antigen-specific effector and memory T- cells migrate to the skin (Figure 1C), inducing inflammation and resultant erythema, spongiosis, or vesicle formation (Figure 1G) (3). While ACD is typically associated with an increased production of T helper (Th)1- like cytokines (4), previous research has demonstrated an increased production of both Th1- and Th2- like cytokines in the peripheral blood mononuclear cells (PBMC) of allergic patients (5).

In addition to immune dysfunction, alterations in the epidermal barrier also play a crucial role in ACD disease progression. Structural proteins of the cornified envelope, such as loricrin (LOR), are essential in maintaining the skin barrier (6). Reduced LOR expression has been demonstrated in tape-strips isolated from ACD patients, suggesting that loss of epidermal integrity may promote ACD progression (7).

Neuroimmune interactions involving two types of skin-resident sensory neurons, peptidergic and nonpeptidergic, further amplify inflammatory reactions and pruritus in ACD. Peptidergic neurons initiate and maintain the inflammatory response by secreting neuropeptides that activate mast cells and promote DC migration and Th2 priming (Figure 1D) (8). On the other hand, nonpeptidergic neurons respond to inflammatory signals upon allergen re-exposure sustaining itch perception (Figure 1E) (8). These interactions form a feedback loop in which continual scratching allows deeper penetration of allergens and sustained inflammation. Understanding these mechanisms is crucial to developing multifaceted therapies that target both immune and neural pathways that govern ACD pathogenesis.

## 3 Current treatments for ACD and limitations

The management of ACD primarily emphasizes allergen avoidance and symptom relief, with avoidance strategies serving as the cornerstone of therapy (1, 3). Lack of skin clearance in response to allergen avoidance for 6–8 weeks should be followed up to evaluate potential exposures and enhance patient education regarding allergen identification and avoidance (3). Topical corticosteroids are commonly prescribed as first-line adjunctive therapy for reducing inflammation in all types of contact dermatitis (CD) (1, 3). Although calcineurin inhibitors such as tacrolimus and pimecrolimus are an off-label use for ACD (3), they offer steroid-sparing alternatives for sensitive areas like the face and eyelids (1). Recalcitrant or severe ACD that is unresponsive to topical therapy may be treated with phototherapy or systemic corticosteroids (3). A limitation of topical treatments is that ACD can occur to the medication's active ingredient or excipients (3).

Recent advances have introduced biologic therapies targeting specific inflammatory pathways. For example, Dupilumab is an IL-4 receptor α- inhibitor that prevents activation of the IL-4/IL-13 signaling cascade, halting the Th2 inflammatory response (9). Though it has been approved for moderate-to-severe atopic dermatitis (AD), the effects on ACD are unclear, with some patients demonstrating ACD improvement on Dupilumab (4, 9). However, despite these innovations, high costs remain a barrier to treatment (10).

## 4 Role of biomarkers in the characterization of ACD patients

### 4.1 Diagnostic biomarkers

Biomarkers hold promise in improving the diagnosis and management of ACD. Although patch testing, the diagnostic gold standard for ACD, does not provide clear distinction from ICD (2), diagnostic biomarkers have been investigated to differentiate the two entities based on immune cell profiles and gene expression signatures. Previous studies of punch biopsies using leukocyte deconvolution algorithms to analyze immune cell composition demonstrated that ACD is characterized by an accumulation of M1 macrophages, natural killer cells, and activated mast cells, while ICD is characterized by increased monocytes and T cells with fewer resting mast cells (2). Functional gene analyses revealed that ADAM8 and CD47 were of greatest importance in differentiating ACD from ICD, which are involved in inflammation and T cell migration, respectively (2). Additionally, the biomarkers CD47, BATF, FASLG, SELE, and IL37 were found to be of diagnostic and therapeutic value in a supervised machine-learning-based approach (2). The roles of these diagnostic biomarkers are summarized in Table 1. Although both ACD and ICD are approached similarly with allergen or irritant avoidance and the use of adjunctive therapies such as corticosteroids, these diagnostic markers must be studied more extensively as they may provide insight into targeted therapies for ACD.

**Table 1:** Biomarkers implicated in ACD and their role in pathogenesis.

Biomarker	Involvement in ACD pathogenesis
ADAM8^a^	• Involved in inflammatory cell recruitment and activation (2)
CD47^a^	• Transmembrane protein that is widely expressed, notably in NK cells (2) • Differentiation of effector cytotoxic T lymphocytes (CTLs) (2)
BATF^a^	• Differentiation of effector CTLs and Th17 cells in ACD (2)
FASLG^a^	• Cell death induction through FAS-FASL interactions promoting immune cell mediated apoptosis of haptenized keratinocytes in ACD (2)
SELE^a^	• Encodes E-selectin (2). Important in T cell rolling and homing (2)
IL37^a^	• Immunoregulatory cytokine expressed by effector memory T cells and inhibits innate immune signaling (2)
LOR^a^^,^^b^	• Key structural protein in the skin involved in skin barrier integrity (6) • Reductions in LOR mRNA have been demonstrated in tape strips of ACD patients (7)
NMF^b^	• Hygroscopic, low molecular weight compounds that promote skin hydration and barrier integrity (6). • Reductions in NMF are correlated with AD disease progression and *S. aureus* infection (6) • Role in ACD is less clear, with some allergens leading to NMF reductions, and others having no effect (13)
TEWL^b^	• Measures passive water flux across the stratum corneum and is positively correlated with skin barrier damage (6) • Can be used to assess the damaging effects of allergens on the epidermis, and monitor response to therapy (6)

^a^ Biomarkers with potential diagnostic value.

^b^ Biomarkers with potential prognostic value.

### 4.2 Prognostic biomarkers

Potential prognostic biomarkers for ACD, including loricrin (LOR) transcript levels, natural moisturizing factor (NMF), and transepidermal water loss (TEWL) offer insights into disease severity and progression. These biomarkers are summarized in Table 1 but are elaborated herein.

LOR is a cornified envelope protein that is implicated in the mechanical and barrier integrity of the skin (6). A previous study by Tam et al. demonstrated reduced LOR mRNA expression in tape strips collected from individuals with ACD compared to healthy skin and ICD- affected skin, highlighting its diagnostic potential (7). Given its function in preserving skin integrity, LOR may also serve as a prognostic biomarker in evaluating inflammation severity, disease progression, and response to therapy.

Other biomarkers such as NMF also play a potential prognostic role in ACD. NMF is composed of hygroscopic, low- molecular weight compounds derived from the enzymatic breakdown of filaggrin, a key structural protein in the in the stratum corneum (6, 11). Decreased NMF levels are a surrogate marker for loss of function mutations in the filaggrin (FLG) gene, which are strongly correlated with AD and impaired epidermal barrier function (6, 11). Additionally, Th2- mediated inflammation has been shown to downregulate FLG expression, leading to subsequent reductions in NMF (6). Clinically low NMF levels may predispose patients to colonization by *Staphylococcus aureus*, which adheres more readily to corneocytes in NMF-depleted skin (12). While studies have demonstrated that reduced NMF levels are correlated with AD severity, the role of NMF in ACD remains unclear. Some allergens have elicited reductions in NMF, whereas others appear to have no effect (13). Notably, several studies have linked NMF depletion to irritants or ICD rather than contact allergens or ACD; however, the irritant properties of certain allergens may contribute to these inconsistent findings (6). Despite these discrepancies, NMF's well-established role in assessing skin barrier integrity and disease severity in AD, coupled with evidence linking low NMF levels to increased susceptibility to *S. aureus* infection, underscore the need for further research in determining NMF's potential as a biomarker for ACD progression and therapeutic monitoring.

Lastly, TEWL is a commonly used biophysical biomarker in dermatological research for its ability to assess skin barrier function. It measures the passive movement of water across the stratum corneum and is positively correlated with skin barrier damage (6). It is also used to assess response to interventions in occupational settings and can thus be a useful in assessing damage to the stratum corneum triggered by ACD and response to therapy (6).

## 5 Neuroimmune interactions and biomarkers involved in ACD-related itch

Neuroimmune interactions contribute significantly to itch in allergic eczema. Interleukin-31 (IL-31) and transient receptor potential vanilloid 1 (TRPV1) appear to be potential neurobiomarkers in ACD, playing essential roles in modulating itch and inflammation (8).

IL-31 is a pruritogenic cytokine produced by activated T cells (14) and mast cells in response to immunogenic stimuli (Figure 1F) (8). NP3 neurons, a subset of nonpeptidergic neurons involved in pruritus, are defined by the expression of the IL-31 receptor (IL-31 R) complex, comprised of IL-31RA and the oncostatin M receptor (OSMR)(Figure 1E) (8). IL-31 signaling activates the JAK/STAT pathway and activates NP3 neurons to secrete brain natriuretic peptide (BNP), a pruritogen that stimulates spinal dorsal horn neurons to propagate itch sensations in AD and ACD (8). Itch is a predominant complaint of ACD that can negatively impact patients' quality of life (15). As such, great attention has been focused on IL-31R inhibition, with inhibitors such as the antibody nemolizumab greatly relieving itch and rash severity in AD studies (8). Given the similar neuroimmune mechanisms of IL-31 in AD and ACD, further studies on the applicability of IL-31 as a marker of ACD- related itch, and its inhibition in therapeutic applications should be investigated.

Additionally, TRPV1, an ion channel expressed on peptidergic neurons (Figure 1D), has been implicated in ACD-related itch in response to allergens such as squaric acid dibutyl ester (8). Interestingly, a newly developed topical TRPV1 selective antagonist, PAC-14028, has demonstrated statistically significant improvements in physician-evaluated IGA (Investigator's Global Assessment) scores in patients with AD, with lower scores indicating greater skin improvement (16). Moreover, although not statistically significant due to limited study sample size, PAC-14028 was also associated with slight improvements in pruritus, SCORAD (Scoring Atopic Dermatitis), EASI 75/90 (Eczema Area and Severity Index), and sleep disturbance scores (16). TRPV1's role in ACD-related itch may thus make it a promising target in alleviating pruritis.

## 6 Discussion

The integration of biomarkers and neuroimmune targets into precision medicine offers new opportunities to better diagnose and treat ACD. Transcriptomic analysis and machine-learning models enable patient stratification, allowing for the identification of biomarkers such as ADAM8, CD47, BATF, SELE, IL-37 that may aid in diagnosis and differentiating ACD from other forms of dermatitis (2). Additionally, LOR (6, 7), NMF (13), and TEWL (6) have been studied as key indicators of skin barrier integrity in ACD and similar conditions like AD. Therapies targeting neurobiomarkers of ACD, including IL-31 and TRPV1 also show promise in reducing ACD- associated pruritus (8).

ACD imposes a great psychological impact on patients, with a reduced quality of life. This can manifest as occupational and non-occupational effects such as anxiety, depression, disabilities that result in inability to perform work activities, sleep disturbances, and limitations in personal, family, and leisurely activities (15). While research on the biomarkers and neurobiomarkers involved in ACD is still nascent, future research should focus on biomarker validation through use of advanced preclinical models (17) and larger clinical trials to better understand their clinical utility and use as potential targets. Future developments may thus allow for multi-targeted approaches that address both immune and neurogenic therapies that enhance long-term ACD control, especially in refractory cases, and lead to the development of personalized care that provides symptom relief.

## References

[1] Nassau S Fonacier L. Allergic contact dermatitis. Med Clin North Am. (2020) 104(1):61–76. 10.1016/j.mcna.2019.08.012 31757238

[2] Fortino V Wisgrill L Werner P Suomela S Linder N Jalonen E Machine-learning-driven biomarker discovery for the discrimination between allergic and irritant contact dermatitis. Proc Natl Acad Sci U S A. (2020) 117(52):33474–85. 10.1073/pnas.2009192117 33318199 PMC7776829

[3] Scheinman PL Vocanson M Thyssen JP Johansen JD Nixon RL Dear K Contact dermatitis. Nat Rev Dis Primers. (2021) 7(1):38. 10.1038/s41572-021-00271-4 34045488

[4] Johansen JD Bonefeld CM Schwensen JFB Thyssen JP Uter W. Novel insights into contact dermatitis. J Allergy Clin Immunol. (2022) 149(4):1162–71. 10.1016/j.jaci.2022.02.002 35183605

[5] Masjedi K Ahlborg N Gruvberger B Bruze M Karlberg AT. Methylisothiazolinones elicit increased production of both T helper (Th)1- and Th2-like cytokines by peripheral blood mononuclear cells from contact allergic individuals. Br J Dermatol. (2003) 149(6):1172–82. 10.1111/j.1365-2133.2003.05750.x 14674894

[6] de Boer FL van der Molen HF Kezic S. Epidermal biomarkers of the skin barrier in atopic and contact dermatitis. Contact Dermatitis. (2023) 89(4):221–9. 10.1111/cod.14391 37571977

[7] Tam I Hill KR Park JM Yu J. Skin tape stripping identifies gene transcript signature associated with allergic contact dermatitis. Contact Dermatitis. (2021) 84(5):308–16. 10.1111/cod.13749 33236775 PMC8026495

[8] Liu AW Gillis JE Sumpter TL Kaplan DH. Neuroimmune interactions in atopic and allergic contact dermatitis. J Allergy Clin Immunol. (2023) 151(5):1169–77. 10.1016/j.jaci.2023.03.013 37149370 PMC10167546

[9] Johnson H Adler BL Yu J. Dupilumab for allergic contact dermatitis: an overview of its use and impact on patch testing. Cutis. (2022) 109(5):265–7. 10.12788/cutis.0519 35856769

[10] Pharmacoeconomic Review Report: Dupilumab (Dupixent): (Sanofi-Aventis Canada Inc): Indication: Moderate-to-severe atopic dermatitis (AD). Ottawa, ON: CADTH Common Drug Reviews (2018). Available online at: https://www.ncbi.nlm.nih.gov/books/NBK539194/ (Accessed January 15, 2025). 30933446

[11] Nouwen AEM Karadavut D Pasmans S Elbert NJ Bos LDN Nijsten TEC Natural moisturizing factor as a clinical marker in atopic dermatitis. Allergy. (2020) 75(1):188–90. 10.1111/all.13942 31179552

[12] Feuillie C Vitry P McAleer MA Kezic S Irvine AD Geoghegan JA Adhesion of staphylococcus aureus to corneocytes from atopic dermatitis patients is controlled by natural moisturizing factor levels. mBio. (2018) 9(4):e01184–18. 10.1128/mBio.01184-18 30108169 PMC6094479

[13] Koppes SA Ljubojevic Hadzavdic S Jakasa I Franceschi N Riethmuller C Jurakic Toncic R Effect of allergens and irritants on levels of natural moisturizing factor and corneocyte morphology. Contact Dermatitis. (2017) 76(5):287–95. 10.1111/cod.12770 28295421 PMC5836858

[14] Niyonsaba F Ushio H Hara M Yokoi H Tominaga M Takamori K Antimicrobial peptides human beta-defensins and cathelicidin LL-37 induce the secretion of a pruritogenic cytokine IL-31 by human mast cells. J Immunol. (2010) 184(7):3526–34. 10.4049/jimmunol.0900712 20190140

[15] Di Agosta E Salvati L Corazza M Baiardini I Ambrogio F Angileri L Quality of life in patients with allergic and immunologic skin diseases: in the eye of the beholder. Clin Mol Allergy. (2021) 19(1):26. 10.1186/s12948-021-00165-6 34930291 PMC8690422

[16] Lee YW Won CH Jung K Nam HJ Choi G Park YH Efficacy and safety of PAC-14028 cream—a novel, topical, nonsteroidal, selective TRPV1 antagonist in patients with mild-to-moderate atopic dermatitis: a phase IIb randomized trial. Br J Dermatol. (2019) 180(5):1030–8. 10.1111/bjd.17455 30623408 PMC6850419

[17] Maskey AR Mo X Li XM. Preclinical models of atopic dermatitis suitable for mechanistic and therapeutic investigations. J Inflamm Res. (2024) 17:6955–70. 10.2147/JIR.S467327 39372589 PMC11456296

